# In/visibly different: Melania Trump and the othering of Eastern European women in US culture

**DOI:** 10.1080/14680777.2018.1546205

**Published:** 2018-12-06

**Authors:** Katharina Wiedlack

**Affiliations:** Department of English and American Studies, Europa-University Flensburg, Flensburg, Germany

**Keywords:** US media, racialization, post-socialist contexts, critical whiteness studies, feminist critique

## Abstract

This article offers a “feminist critical discourse analysis” of the *Saturday Night Live* sketch “Melanianade.” It argues that the comical video reinforces and essentializes negative stereotypes of Eastern European women to depict Melania Trump, seeking to delegitimize white hegemonic masculinity and female complicity. The fictitious Melania Trump’s appearance needs to be understood in her co-construction with her white hegemonic husband and otherwise racialized women in the comedy sketch. These women are the African-American women of Beyoncé’s video “Sorry,” which “Melanianade” copies/satirizes. “Sorry” represents Black female US-American experience, and was broadly understood as Black feminist art/activism. Taking Beyoncé’s place in the video, the fictitious Melania Trump is co-constructed to the absent black feminist bodies as white non-feminist Eastern European Other. Using Beyoncé’s video as a template, “Melanianade” re-affirms a discourse of Otherness that re-establishes the enlightened and emancipated educated (white) feminist American non-immigrant woman as norm, while it also whitewashes the Black American experience, which “Sorry” stands for.

## Introduction

This article offers a feminist discourse analysis (Michelle M. Lazar 2007) of the *Saturday Night Live* (*SNL*) sketch “Melanianade.” I argue that, as the mainstream feminist video seeks to delegitimize white hegemonic masculinity and female complicity, it reinforces and essentializes negative stereotypes about Eastern European^1^ women to depict Melania Trump (MT) in the process. Following post-socialist accounts on the *Othering* and *orientalizing* of Eastern European women (Larry Wolff 1994; Madina Tlostanova 2015) I argue that MT’s appearance as essentialized *white Eastern European Other* needs to be understood in her co-construction with her hegemonic husband and otherwise racialized women in the comedy sketch. These other racialized women, who are not directly represented, are the African American women of Beyoncé’s video “Sorry,” which “Melanianade” copies and satirizes. “Sorry” represents female Black US-American experience, and was broadly understood as Black feminist art/activism (Jessica Opatich 2016; Andrea Waguespack 2016). In “Melanianade,” which aired first on NBC’s *SNL* on October 16 2016, the white American actress and comedian Cecily Strong imitates MT, mimicking almost completely Beyoncé Knowles-Carter’s performance, poses, and dances in her highly acclaimed music video “Sorry,” diverging only through different lyrics and pronunciation from the original. Taking Beyoncé’s place, the fictitious MT is co-constructed with the absent black feminist bodies as *white* non-feminist *Other*. Using “Sorry” as a template, “Melanianade” affirms a discourse that muddles different forms of *Otherness* into one, while confirming the enlightened, emancipated, educated (white) feminist American non-immigrant woman as norm. Moreover, as the video uses the well-known song and video to mark MT as *Other*, it whitewashes the Black American experience, which “Sorry” communicates.

The black and white video “Melanianade” opens with MT’s narrative voice, in a heavily accented English (emphasis on the R, pronounced with a rolling sound): “Here lies my last nerve, Donald.” The video represents MT as “beautiful, dutiful,” mirroring media representations, calling her strikingly “passive” (Guy Trebay 2016, D1), “in an embrace of values from an era when a potential first lady might be less likely to have served as her husband’s former law firm mentor (as Michelle Obama once was) than his carpet ornament” (Trebay 2016, D1). The following scene shows the fictional Ivanka (Emily Blunt) and Tiffany Trump (Vanessa Bayer), Kellyanne Conway (Kate McKinnon), and Omarosa (Sasheer Zamata), without a last name, sitting lined up in a moving vehicle. This scene introduces all the characters of the video—the silent wife; the daughters; the spokesperson Conway; and the Director of Communications for the White House Office of Public Engagement and Intergovernmental Affairs and former star of Trump’s Reality TV-show *The Apprentice*, Omarosa Manigault.^2^ Additionally, the scene makes a very visible reference to the opening of Beyoncé’s video “Sorry.”

“Sorry’s” story evolves around Beyoncé’s betrayal through her husband. Additionally, it tells about female Black American history, from slavery and coerced domestic servitude, to contemporary systemic gendered oppression. It starts with Beyoncé, reciting a spoken-word piece by Warsan Shire, the Somalian, UK-based poet, accompanied by the slow jewelry box melody of a theme from Tchaikovsky’s ballet *Swan Lake* (Waguespack 2016; Opatich 2016). The poem’s line “So what are you gonna say at my funeral, now that you’ve killed me?” speaks metaphorically of a husband’s infidelity from the perspective of the betrayed wife; but it also comments on systemic gendered violence against Black women. The scene is set in a public bus. Black women are sitting on bus benches with ceremonious painted faces, called “Sacred Art of the Ori” (ABC News 2016), “inspired by Yoruban rituals and designed by Nigerian-born, Brooklyn-based Laolu Senbanjo” (Lisa Perrott, Holly Rogers, and Carol Vernallis 2016). They perform modern dance movements collectively, which highlights their ceremonious face-paint and makes them appear as one moving body, rather than individual dancers. Their identical black shirts and trousers as well as the public bus are references to the Black Power movement and the Civil Rights movement, and Rosa Parks’ act of resistance. Little flashes of light illuminate the metal roof of the bus, bringing to mind star showers as much as the metal rods of prison cells, as the movement of the bus distorts the little light dots into stripes.

The women in “Melanianade,” in contrast, are all recognizable as individuals as they sit in a limousine. The same little flashes of light circle the vehicle, but they illuminate not raw metal, but a comfortable, richly decorated interior. The women wearing Manolo Blahniks^3^ and other classic fashion items such as black and white slim-fit dresses, and pussy-bow blouses,^4^ represent upper-class individuality. Their ritualistic body movements copy the performers’ of “Sorry,” but their references, beyond being a reminder of Beyoncé’s iconic piece, are empty.

Already the first scenes of “Melanianade” bring up the popular US-American discourses about MT, which the scriptwriters of the *SNL* video arguably built on, affirming some of their crucial ideas. I read the significations and meanings of MT in “Melanianade,” drawing out the stereotypes along which her figure is modeled. Addressing the specific forms of othering of MT within “Melanianade,” I aim to show how the *US-gaze* constructs Eastern European women as mentally, ideologically, morally and ethically, but also bodily different to US-American non-immigrant women. I build my arguments on the work of the Gender Studies scholars Kimberly A. Williams (2012), Anca Parvulescu (2014), Valentina Glajar and Domnica Radulescu (2004), and Agnieszka Tuszynska (2004), analyzing the cultural representations of post-socialist women within Western media and culture. I push their arguments further, by focusing the othering of female Eastern European bodies through whiteness.^5^ Building on Claudia Sadowski-Smith’s (2018) finding that US-American popular culture imagines post-socialist immigrant women as homogeneously white, I understand MT as *white Other* to the US-American white norm as well as to racialized American immigrant and non-immigrant bodies. I follow Williams (2012), Parvulescu (2014), Glajar and Radulescu (2004) as well as Tuszynska (2004) in locating the othering of Eastern European female bodies on the intersection of sexualization and class-occupational constructions. They show that the signification of Eastern Europeanness muddles women of varied heritage or descent into one category, by extinguishing the differences between them, and heightening the difference/inferiority to the Western norm. Parvulescu examines in most detail how Eastern European women’s bodies emerge through their sexualized class location. Although her analysis is useful to explain how MT emerges in comparison to and relation with otherwise racialized US-American women and white hegemonic US-American men, I diverge from Parvulescu in labeling her specific cultural location as “not-quite-white” (2014, 14). On the contrary, I argue that MT’s whiteness is a significant meaning; however, one that does not automatically allow her to join the US-American white norm. Her whiteness is more than just a “passing” (Parvulescu 2014, 14). Although recognized as white, much like white working-class people, MT is not recognized as authentic part of the white American elite. Rather, she is a cheater or “trickster” (Glajar and Radulescu 2004; Tuszynska 2004), who illegitimately holds a place of power, due to her Eastern European heritage. While her whiteness and beauty bear the potential to blend in, to pass as something that she is not, her sexualization signifies her *Otherness* through style and language/pronunciation. Understanding her as victim of her husband’s toxic masculinity further guarantees that she stays “arrested” in the position of Eastern European under-classness.

I read these findings against the works on the geo-temporal and developmental location of Eastern Europe within the heritage of Western colonial Orientalism and discourses on ethnicity/race.^6^ Cultural studies researchers such as Wolff (1994), Robert Kulpa and Joanna Mizielińska (2011) or Tlostanova (2015) have long established the idea that the signification of Eastern Europe and especially its female population was designed within the project of the European Enlightenment to legitimate and further establish its Orientalism, to claim and essentialize superiority over racialized Eastern and Southern people. This signification of people and bodies between the poles of Western European civilization and Southern and Eastern barbarianism assigned Eastern Europeans to a geographical and temporal (developmental) gray area of the *in-between*. Combining the feminist findings on essentialized stereotypes of Eastern European women with the work on their geo-temporal location allows me to understand their construction as in relation to the bodies they co-appear with on a material and geo-temporal level. It allows me to understand their in-between position as construction of a yet unreached potential in Eastern Europeans to become what Western Europeans and white US-Americans have already become. They signify the white raw material that needs to be formed according to Western standards.

Following this idea, I put the emphasis of my reading on the relational construction of MT in “Melanianade.” I argue that the readability of Eastern European *Otherness* depends on the co-appearance of the poles between which it is signified. In opposition to racialized African Americans, Eastern European immigrants to the US can appear as desired white bodies. Yet, in the presence of white sophisticated American women, Eastern European women appear as their “lesser cousins” (Kulpa and Mizielińska 2011, 17). “Melanianade” constructs MT visually and verbally as white *Other* to white modern American feminists as well as Black feminists. Ironically, it does so by appropriating Black American feminism and whitewashing or relieving it of its Black Power criticism in the process. Contrasting “Melanianade” with its model—the Beyoncé’s video “Sorry,” and its meanings—highlights the satire’s dependence on the cultural leverage of this music video. In analyzing Black female US-American experience as key aspects of “Sorry,” I show the interdependence between Melania’s signification as Eastern Europe *Other* in “Melanianade” and the signification of Beyoncé’s latest work as Black feminist art/activism.

## Stereotypical notions of Otherness

*SNL*, as many critics have pointed out (Dean Obeidallah 2017; Joanna Robinson 2017), is not only fighting the “American Culture War”^7^ (Stepehn Prothero 2016) on the side of the liberal democratic elite against right-wing conservatism, it is also satirically reflecting the whole process. The othering of MT is part of the fight for cultural hegemony. Ironically, *SNL* thereby reaffirms conservative ideas of a superior national culture that depend on the sexualizing, gendering, and racializing of the Eastern European *Other*. Like conservative political and cultural commentators, *SNL* portrays MT as uncharismatic doll and “trophy wife” (Jelena Prtoric 2017), drawing on misogynist and anti-immigrant views, echoing neoliberal media, such as *The New Yorker* (Lauren Collins 2016), which described her as “the perfect body on which to hang a brand.” *The New York Times* reporter, Jacob Bernstein, even referred to MT publicly as a “hooker” (Katie Yoder 2017).^8^ Feminist discourses present MT as “a victim of her husband [and] a submissive tool” (Prtoric 2017). Under the “#FreeMelania” hashtag (Weaver 2017; Jill Filipovic 2017; Lizzie Crocker 2016) they speculated that MT might be the victim of domestic abuse. “But if Melania is a victim,” as Filipovic (2017) rightly pointed out, “a cheeky hashtag belittles her situation. And considering that we don’t actually know she’s a victim at all, positioning her as a helpless pawn rather than an adult woman is deeply condescending.”

These examples of sentiments against MT show how mainstream discourses see her as the embodiment of an elite version of two very popular, not mutually exclusive stereotypes of post-Soviet women, the slut and the victim. The media studies scholar Roumiana Deltcheva identified the post-1991 most common filmic depictions of Eastern European women: “the scrupulous slut, the conniving trickster, and the helpless victim” (2004, 164). Each of these stereotypes, which all go back to the virgin/whore dichotomy, “carr[ies] distinct negative connotations that, in their totality, reinforce the idea of Otherness as negation: negation of voice, negation of space, negation of experience” (Deltcheva 2004, 181). Ignoring her multilingualism, and status as elite earner as supermodel, commentators make fun of her accent and suggest that she needed to sell herself for money (i.e., as “hooker” or sex worker) (Eric Andersson 2016). Instead of focusing on her as a businesswoman, reports present her as bound to domesticity, and focus on her body modifications and modeling past, to construct her as mere trophy wife (John Aravosis 2017). These discourses create the epistemological framework for the script and materialization of “Melanianade,” and made it understandable in a liberal progressive pro-feminist context. They draw on the long tradition of classifications of Eastern European bodies as in-between the two poles that span a hierarchical system of significations along the threads of race/ethnicity, class/occupation, gender, sexuality, and age (Wolff 1994; Williams 2012), as the site at which global East and West “confront each other” (Tuszynska 2004, 204). They render Eastern European woman as “a commodity” (Tuszynska 2004, 204) and as similar, yet different *Other*, and their bodies as in “limbo,” built to demarcate an US-American identity. The emphasis on motherhood and sex work (Yoder 2017) are two ends of the same continuum (Parvulescu 2014). Both labels signify Eastern European women through notions of reproductive labor, and sex work/trafficking on a hierarchically lower position than white non-immigrant women and mark their difference to the latter as racial *Otherness*. As such, the Eastern European body of MT can be used on several occasions to mark or negotiate an opposition to the superior enlightened US-subject. Discursively constructed as site and commodity of East/conservative West/liberal value negotiations, she becomes used most frequently to construct a positive notion of the US-national progressive, liberal, feminist “us” in opposition to the misogynist and racist husband Donald Trump. Tuszynska identifies the oppositionality between white heterosexual affluent (older) men, and the beautiful younger white Eastern European women as one common dichotomous co-constructions. Another one is the African American woman in opposition to the Eastern European woman.

Before further analyzing the location of Eastern European women within the epistemology of ethnicity and racialization in the US on the example of “Melanianade,” I want to briefly come back to the suggestion that MT is a victim of domestic abuse. The depiction of Eastern European women as sexually exploited victims has a long tradition in the US. The US Trafficking Victims Protection Act was strongly influenced by a public case of white female Russian and Ukrainian trafficking victims in 1997 (Williams 2012, 94). As result of Western anti-trafficking activism that has built on “a melodramatic narrative” of female Eastern European naïveté, economic disadvantage (backwardness), and victimization, the US law became the template for international work and legislations (Williams 2012, 94). It crucially emphasized the US’s self-perceived role as leader of the free world and human rights advocate. This self-fashioning of a positive progressive feminist US identity by commodifying vulnerable white female Eastern European bodies (of victimized sex workers) is another important aspect of the framework to understand the visual language of “Melanianade.”

## “Melanianade” or the tenaciousness of stereotypes

“Melanianade,” staying true to its comedy format, brings forward a very blunt feminist critique. Cecily Strong plays MT on the brink of feminist emancipation. “I can’t take it anymore,” she utters with a saddened face in the opening sequence of the video, and Ivanka, Kellyanne, Omarosa, and Tiffany echo: “you’re breaking us, taking it for granted that we’ll always be there.” They speak to President Trump’s misogyny, to his sexism and racism. They criticize him by imitating a video that has been praised as Black feminist empowerment video (Inna Arzumanova 2016, 421–424). Arguably, the celebration of Beyoncé’s video as Black feminist politics was the reason why the *SNL* writers Chris Kelly and Sarah Schneider chose to use it as a template. (Beyoncé’s usage of the music box version of *Swan Lake*, an Eastern European/Russian cultural product, might have been another reason.) In any case, the widely disseminated cultural knowledge about the feminism of the song and video “Sorry” and its album *Lemonade* heightens the feminist critique on Trump’s misogyny in “Melanianade.” Additionally, and maybe unintentionally, in using “Sorry,” a piece of art that signifies Black feminist politics, to mark white ethnicity and gender politics, sexualization, and victimization, shows the reference from ethnicity to race, and the damage it does. It highlights the signification of the real existing MT as *Other*, through the strong reference to African American women and their experiences and history. Through this process of referencing, “Melanianade” whitewashes the referenced subjects and transforms the meaning of a specific Black female embodiment into a more general *Otherness*. To put it differently, Beyoncé’s video illustrates the corporeal violation but also resistance of African American women; in using the same imagery to signify oppression against white women, the parody “Melanianade” highlights MT’s body in the video, as a somehow different and oppressed body, yet it silences the discussion of color-based racism, which the original brought forward so forcefully.

MT is presented as embodied commodity, a docile wife to a man embodying white toxic masculinity. She is the commodity of the white American man in opposition to the American feminist subject, invoked as invisible norm. In reference to the Black feminist Beyoncé, whose music video “Sorry” arguably most viewers see behind the satire, MT appears as white, but non-American. The topic of *Otherness*, gendered, sexualized, culturalized, racialized difference forms the filter or gaze, through which the (US-American) audience watches the video. It presents MT as a “beautiful, dutiful” wife, activating the stereotypes of the silent and devoted Eastern European woman (victim), banned from the public sphere, oppressed by her dominant husband. “Coming from the presumed ‘desolate setting’ of post-communism …, eastern European women are usually seen as particularly docile and submissive in the eyes of Western men. Besides that, eastern European girls still have the reputation of going weak at their knees for western men.” (Jelena Prtoric 2017). Yet, being the “beautiful, dutiful” wife, her embodied difference is neither a Black feminist, nor a white feminist.

## Racializing MT’s whiteness

Because MT is marked as different to the rest of the “Trump women,” through her accent and partly her outfits and accessories, the signification of *Otherness* sticks to her body, arguably even more than to the Black, but upper-class American Omarosa. MT’s physique, her beautiful body and hair become pronounced through the technique of the filming. The camera focus from slightly below draws attention to her cleavage and long uncovered legs, her skin and hair appear smooth and without blemish through the lightening in black and white tones. What additionally accentuates MT’s body in “Melanianade,” besides the obvious title, is the knowledge about Beyoncé’s video.

“Sorry” represents Black women. In the original, Beyoncé sits cross-legged on a stage, wearing a hairstyle reminiscent of African art and the work of the model and singer Grace Jones. Beyoncé’s references—the different hairstyles, clothes and costumes, surroundings and stages—are densely conjured links “to black liberation and practices of radical black resistance [and] legacies of black spirituality” (Arzumanova 2016, 421). The facial paintings, wardrobe made of fabric with ethnicized patterns, references to African and Caribbean culture, and the cross-legged Beyoncé are strong cues to the racialization and cultural heritage as well as culturalization of African American women. In “Melanianade’s” mirrored image, MT equally sits cross-legged in the middle of a stage, wearing a big fur hat, also making a reference to ethnicity, reminding the viewer of a Russian Ushanka. The line “I have an Eastern European mind set—I forgive but don’t forget” further emphasizes the ethnicized hairdressing or, rather, ethnicity as such. While Beyoncé’s self-depiction honors African culture or heritage, MT’s stereotypical Eastern European hat simply marks her as *Other*.

In comparison to Beyoncé’s black feminism, MT’s whiteness “sticks out,” becomes highlighted. Public knowledge about Beyoncé’s nickname “Queen B” and the interpretation of her strong feminine agency and power, as being carried out with the attitude of a female ruler or monarch, additionally influence the reading of MT. MT is depicted, much as Beyoncé in “Sorry,” as queen. In her case, however, this does not signify rebellion and agency, but etiquette and superficiality. Since the video connects MT, a white Eastern European woman, and Beyoncé, a Black African American woman, the category of race lingers behind the feminist critique it proposes. Yet, through the non-verbalization of the racialization of Eastern European women or Donald Trump’s racism, it renders the discussion of racism impossible and arguably whitewashes Beyoncé’s Black feminism at the same time. The weak attempt to highlight the intersection between President Trump’s misogyny and racism, by presenting Omarosa as a woman without a last name, addressing the linguistic hegemony and the unwillingness to “learn” non-normative names, does not sufficiently irritate the process of making Black oppression invisible. Thus, the positioning of MT as queen highlights her Eastern Europeanness, bringing up a broader cultural reference from immigration to a long-gone European monarchy. Williams (2012) and Sadowski-Smith (2018) point to the cultural knowledge that imagines Eastern European women as descendants of long-gone but glorious and mysterious royal families. Deriving from mythologized places, Eastern European immigrant *Others* are imagined as white, but their discursive attachment to the Eastern European (royal) past prohibits them from becoming fully modern progressive US citizens.

Most importantly, the focus on MT’s body as replacement of Beyoncé’s and in itself shows her construction as white racialized Eastern European *Other*. The critical tools available within feminist or critical race studies to account for the racialization of white Eastern European women are limited. I follow Stuart Hall, who conceptualizes race as a discursive construct, “principal of classification,” and “a sliding signifier” (Stuart Hall 1997). Using race and racialization to describe and analyze representations of Eastern European bodies equally makes the processes at play visible as it produces these bodies as racially specific. Although the signifier race is unstable and in flux, its meaning is not arbitrary or random. At the heart of the concept of the “Slavic race” rests the already frequently mentioned orientalist design of Eastern Europe “as a world ‘over there,’ an alien world of differences that is light years away not only from the economic prosperity but also from the social conventions and values of the West” (Deltcheva 2004, 162). The most common criticism of racism within the US focuses on the construction of color. Yet, racism “targets East Europeans on the basis of markers that are not limited to color” (Parvulescu 2014, 15). The “stratification insignia” (Hall quoted in Parvulescu 2014, 15) that “stick” to Eastern European women are “racialized physical characteristics like hair, teeth, body type, and clothing styles as well as education, religion, and values” (Hall quoted in Parvulescu 2014, 15). Moreover, I argue that the racialization of Eastern European women is best framed as materialization of the in-between of two poles, the differentiation between nature and culture, between the raw flesh of the barbarian *Other* and the sophisticated intellect of the civilized individual. The racialized focus on MT’s embodiment appears clearly in the caricaturist impressions of MT, by model Gigi Hadid “ducking her lips and mocking Melania’s eastern European accent” (Prtoric 2017), but also in the video “Melanianade,” where the male gaze of the camera accentuates MT’s body parts—her long hair, long legs, spotless white skin, always slightly puckered lips, emphasized cheek bones etc.—forcing the audience to pay attention to them. This male gaze is partly ironic, partly a maybe unintentional result from copying the aesthetic of Beyoncé’s video. It is meant to criticize that MT seems to be “just ‘an object’ to her husband” (Will Worley 2017), that “[t]hese queens in the House of Trump—… models, arm candy, reality-show stars, humiliated sidekicks and shopping channel mavens—are vestal virgins in the temple of acquisition” (Nina Burleigh 2017). Yet, while pointing out President Trump’s sexism, the media uses severely derogatory language to describe the women themselves, and simultaneously reduces them to victims. Especially in the case of MT, the press furthermore legitimizes a medial gaze on her body and character that reminds readers of scientific practices using magnifying glasses, scales, and other surveying instruments to highlight her physical characteristics, and her Eastern European heritage.

Although the usage of the term “racialization” seems appropriate to signify the embodied significations of female Eastern Europeanness, it needs to be emphasized again that such a denomination must not be confused with the signification of skin color. Eastern European women are often signified explicitly as white. Their whiteness is central to their racialization. The whiteness of European, “Slavic” women makes them “a favorite and convenient site for the accumulation of stereotypical images feeding Western lust for the exotic and fear of the ‘barbaric’ ” (Glajar and Radulescu 2004, 162):

[They] are not drastically Other and thus are endowed with an aura of familiarity, or Europeanness, and yet they are not fully familiar or European either, as they come from the more remote regions of Europe, perceived as almost Oriental, as almost exotic, yet not fully so. (Glajar and Radulescu 2004, 162)

MT’s physical appearance, according to news media as well as the video “Melanianade,” complies with American beauty standards. Yet, her embodiment of the American norm is viewed as artificial, exaggerated, as “trying too hard.” She wants to trick the viewer into believing she is what she is not. Her Eastern European mind-set differentiates her from the “better, more tolerant nation” of the US that was formed by “the women’s movement” (Burleigh 2017). She is viewed as backward, domestic and superficial, a “young and beautiful piece of ass” (Burleigh 2017), and, most importantly, as belonging to the class of the “newly rich immigrant.” She is the “ultimate reality-show [star,] impressing Donald Trump, his fellow oligarchs and captains of supranational corporations with [her] looks and poise” (Burleigh 2017). Her life is further called “surviving” and a loss of dignity is suggested. We can identify the signification of MT as a hierarchically “lesser” white woman with Bridget Anderson (2000) and Parvulescu (2014) as essentialized class-occupational stratification. The focus on her body in connection with her prior occupation as model presents both aspects as natural or belonging to her heritage as Eastern European. Her class-occupation as (former) working-class model (suspected sex-worker) in connection to her body appears as ethnic/racial markers, without being identified as non-white skin color.

## Appropriating Black music or a failed emancipation

In many ways the political comedy show *SNL* is a pedagogical project, showing through satire the wrongs of the American society and political elite. Following this idea, the show lets the fictitious MT in “Melanianade” “speak up” in an attempt to emancipate herself. Twisting Beyoncé’s provocative question to her cheating husband, and the US mainstream that discriminates African American women, “Are you sorry,” into “I’m not sorry,” the fictitious MT throws a “Donald, No!” at her husband. Her emancipation is not only signified by her speech, but also by the usage of Beyoncé’s music. “Sorry” is an electro-Rhythm and Blues (R&B) song, with a particularly thumping beat created by synthesizers, drums, and bells. Beyoncé’s self-presentation, her clothes, and the dance performances correspond with the genre of R&B, which is generally consider a Black music genre, but also opens it up for other Black artistic expressions and conventions, as already described. Especially powerful is Beyoncé’s feminist usage of the dress and style conventions of R&B, which is usually considered to be a rather misogynistic genre that objectifies women (C. M. Frisby and J. S. Aubrey 2012). Beyoncé reclaims the sexualized conventions of the genre through her strong powerful feminist attitude. MT’s presentation in “Melanianade” tries to mirror her move, by equally appropriating sexually connoted tight clothes. Copying Beyoncé’s bold poses is meant to signify more of her emancipation, her becoming a (Western-style) feminist.

We can understand the feminist pedagogical project of “Melanianade” with Kulpa as “leveraged pedagogy” (Robert Kulpa 2014, 432). Kulpa developed his concept to explain Western strategies of sanctioning or disciplining Eastern European misogyny and homophobia as “didactical and cultural hegemonic relation of power,” where the East “figures as an object of Western pedagogy” (Kulpa 2014, 432). MT’s complicity in her husband’s misogyny and xenophobia are the content of the comical lecture of “Melanianade.” Her Eastern Europeanness signifies simultaneously her nature, a state of being not-yet-emancipated, since the East only oppresses women, producing “beautiful, dutiful” females, and “docile wives,” as well as her excuse for participating in her husband’s wrongs. The “leveraged pedagogy” of “Melanianade” sanctions her misbehavior with mockery and ridicule that is meant to “teach” Melania emancipation. She should free herself, by standing up for herself, mimicking Beyoncé’s act of showing her unfaithful partner the middle finger in a powerful collective dance performance. Replicating the pedagogical doctrine, supposed to transform MT into a modern emancipated woman, “Melanianade”’s lyrics also use leveraged pedagogy towards the fictitious Donald Trump. MT’s emancipatory performance threatens her husband with leaving him, if he does not stop misbehaving, reminding him that without her and her female co-performers he would not be in the presidential seat.

Yet, the emancipation of MT, in contrast to Beyoncé’s rebellious act of rejecting misogyny and sexism in “Sorry,” does not succeed. “Melanianade” is not a funny taking-up of a feminist emancipatory project, because copying Beyoncé’s powerful appropriation of R&B cannot work from the position of a white woman. It only cleanses the musical piece of its original anti-sexist, emancipatory gesture, thereby even killing its own punchline: the appearance of Alec Baldwin as Donald Trump at the end of the video, commanding “his women” into docile obedience, is not funny, since you cannot stop a feminist riot that has never started. Rather than signifying agency, the video’s mimicking of R&B music and style re-establishes the sexualization of its female protagonists and recreates the narrative of MT as typical Eastern European femme fatale. Eastern European women have often been portrayed as cunning femmes fatales (tricksters) instrumentalizing their “deceptive sexuality” (Williams 2012, 36) or “slutiness” (Deltcheva 2004, 181) to lure Northwestern men into their web, to steal their money or power. In other words, MT’s awakening can easily be read not so much as feminist emancipation, but as showing her true (Eastern European bitchy/trickster) side, luring the viewer into her artificial world.

Although the criticism of this world—the bombastic capitalism of the Trump dynasty—needs to be acknowledged, the strategy with which “Melanianade” brings forward such critique is highly problematic. Sketch writers Kelly and Schneider exchange Beyoncé’s setting—the places of historical and contemporary oppression, the public bus, the Southern mansion and plantation—with a stretch limousine and the Trump Tower. Showing an Eastern European ex-model in an over-decorated gold palace is not nearly as subversive as showing a Black female hip hopper taking control of an antebellum mansion (as “Sorry” does). There is nothing provoking or subversively irritating in showing MT in an environment that signifies wealth and European heritage, sitting in a rococo chair surrounded by marble and chandeliers. On the contrary, it is confirming what journalists and commentators suggest between the lines, when they emphasize the large age difference between the spouses, for example (ETN 2013; Worley 2017; Burleigh 2017); namely that MT married Trump out of expediency and for his money, again conforming that she is not only a slut, but also a trickster. The signification and exotization of MT as Eastern European is further shown in another scene copied from Beyoncé’s video “Sorry.” Connected to the choice to locate “Melanianade” in the New York City Trump Tower to critique the newly rich pomp, extravagance, and wastefulness is the choice of attire. “Melanianade” exchanges Beyoncé’s strong sassy Black feminist’s body-positivity for a mix of the conventional style of upper-class white American conservatives and the exuberant in-your-face splendor we know from popular figures such as the Kardashians or Paris Hilton. The decoration of MT with diamonds, fur, and sexy designer dresses and accessories (“Gucci”) conforms to the stereotypical depiction of Eastern European women as sexy “bitches” or sluts once again. The references to European labels and style are intended to mock Donald Trump’s public announcements to boost the American economy and American products, while his wife continues to buy European fashion (Kate Dwyer 2017; Vanessa Friedman 2017). This form of mockery furthers the already strong depreciation of women in general and Eastern European women in particular as superficial, and indeed artificial, attention-hungry Barbie dolls.

## Essentializing the “in-between”

Before closing this article I come back to my argument that Eastern European othering needs to be understood as a form of racialization in a relational geo-temporal context. MT’s body and persona, as already established, appear in “Melanianade” as distinctly different to the African American bodies of its model “Sorry”. Since Beyoncé is not just an African American R&B singer, but also a very popular Black feminist, MT’s *Otherness* needs to be understood as doubled: she is the *Other* to African American bodies, as well as to the highly progressive (developed) US-American feminist intellectual. This *Otherness* can be analyzed by Kulpa and Mizielińska (2011) as signifying her stereotypical Eastern European “lateness” or “unoriginality.” As an Eastern European woman, she can only be seen as a copy of the developed and liberal. A significant clue to Melania’s construction as “latecomer” to modernity and progress is her last sentence in “Melanianade,” shortly before she and the other women obediently and quietly follow Alec Balwin, as Donald Trump, out of the room: “I wrote that all by myself.” This sentence addresses the real MT’s first public speech at the Republican National Convention during Trump’s campaign for president. The speech was in large parts plagiarized from Michelle Obama’s Democratic National Convention speech in Denver in 2008 (Gregory Krieg, Eric Bradner, and Eugene Scott 2016; David A. Graham 2016). While many of the comments following the speech were sexist and derogatory, the *Washington Post* stood out by running an article that explained Melania’s plagiarism with “the culture of cheating in eastern European schools” (Monika Nalepa 2016). According to the journalist Monika Nalepa, MT’s penchant for plagiarism is a result of the communist-era educational system that emphasized memorizing rather than individual thinking. In an open letter to the *Washington Post* published in the *Balkanist*, the North American scholars Irina Ceric, Ana Grujic, Jasmina Tumbas, and Bojana Videkanic (2016) were the first to strongly reject and uncover the culturalist sentiment behind such a claim. Feminist voices from within Eastern Europe equally criticized this stereotypical claim (Agata Pyzik 2016; Prtoric 2017), understanding MT’s defamation as product of the uncreative post-communist East as the peak of more general derogatory discourses that project stereotypical characteristics onto the current First Lady. The view on Eastern European people as unable to produce original thinking, and accordingly stealing from their Western peers, does not only confirm their questionable morals and character, it also confirms the superiority of Western (here US) thinking, culture, and being. This sentiment is just another version of the previously identified trickster stereotype. Mirroring this essentialized character trait of Eastern Europeanness at the level of sound, music, and visual art, “Melanianade” copies Beyoncé’s song “Sorry.” Involuntarily, it exploits the cultural signification of Beyoncé’s album and song as a comment of racialization and racialized oppression to enhance the visibility of MT’s ethnicity, as a comic relief. At the same time, the piece does not explicitly talk about or criticize the common exoticizing and racialization of MT, thereby rather reiterating the oppressive mechanisms and at the same time whitewashing the Black critique of Beyoncé’s art. The fact that the video is intentionally feminist and carries the connotation of feminist critique supports the interpretation of its political correctness, hence the ignorance towards the video's racism.

## Conclusion

Reading the *SNL* clip “Melanianade” as a satirical version of more general media representations of MT, I analyzed the specific forms of othering and racialization as white Eastern European women in this article. In the short comedy video we can see clearly how political discourse engages with stereotypical narratives around Eastern European women to delegitimize not only or primarily MT, but rather the powerful white men she becomes co-constructed with. The parody is a rich example of multiple different aspects of othering Eastern European women: it others MT through her accented speech, hairstyle, attitude, the look in her eyes, and the way she curls her lips. Her clothing style, sexualized body performance, and accented language are imbued with racial meanings through their connection to specific occupational positions (sex work), immigration discourses, and discussions on dishonesty or fraud (trickster identity and plagiarism). As representations of difference, these aspects of physical or bodily differences can be understood as racialized. The processes of racialization, however, become understandable only in a reflection of the videos mimicking Beyoncé’s video “Sorry.” The song and video “Sorry” are interpreted as thematizing Black American history, and feminist African American culture, as part of Beyoncé’s “coming out” as Black activist. Closely analyzing the comedy video “Melanianade” in comparison and relation to “Sorry,” I have shown that the meanings of “Sorry” are as significant for the understanding of “Melanianade” as the latter’s content, sound, and visuals. In focusing on the representation of the fictitious MT in contrast and relation to Beyoncé in her video “Sorry,” I have argued that the racialization of Eastern European women can best be accounted for as “in-betwenness” by understanding their locatedness in connection to hegemonic white men and women of color on the intersection of gender, class, age, origin, language, and sexuality. I have further shown that contemporary racializations of MT as Eastern European woman go back to ideas of development and civilization. Following these ideas, Eastern European women are dignified with exotic (old-fashioned) femininity, and constructed as desiring (and confirming) the domination of Northwestern men. The appeal in their exoticism lies particularly in their similarity (including whiteness) to those who create and direct their invisible/visible *Otherness*. Sexualizing MT, signifying her *Otherness* through style and language/pronunciation and understanding her as cheater/trickster, slut, and victim of her husband’s toxic masculinity, makes sure that she stays “arrested” in the in-between of Eastern Europeanness. In other words, as white woman, marked through a difference not only to other racialized women, but also white American-born women, MT is assigned to the class of the almost-but-not-quite-yet accomplished progressive and modern working women. The gesture behind “Melanianade” is clearly a feminist one, trying to use and show leveraged pedagogy, to lovingly bully the real MT into her own emancipation and make her want to change her husband. Unfortunately, by appropriating the video “Sorry,” and building on its signification as a stronghold of Black feminism, the video whitewashes the same Black feminism, by leaving out a critique of racialization and race-based violence, and essentializes the racialization of MT as Eastern European *Other*.

## Notes

1. The terminology of “Eastern European” is used to make the construction of bodily and character differences visible. It participates in the construction of difference and othering, as much as it deconstructs it. It muddles white citizens from East of Slovenia’s border with Italy to West of Russia’s shore at the Benign Sea into an undistinguishable exoticized mass of people that allegedly share some very specific, often contradictory physical and mental characteristics. Using such a messy and imprecise label, I aim to highlight the violence of simplifications and categorization, intending to fight its oppressive forces, and not increase them. Hopefully, I open a critical discussion of the violence involved in the racialized and sexualized cultural construction of Eastern European women.

2. By the time this article is published, Kellyanne Conway has been fired as spokesperson and Omarosa Manigault is no longer Director of Communications.

3. Expensive, tall high heels designed by the Spanish designer Manolo Blahnik that arguably have become some kind of an upper-class status symbol much like other designer labels such as Chanel or Dior.

4. Especially, the pussy-bow blouse signifies American high-end fashion. Its emergence is connected to prestigious designers such Coco Chanel and Yves Saint Laurent (Stella Bruzzi 2012, 49).

5. “Whiteness” is not an essence, natural, or even a color, or fact that can be proven, seen or identified. Rather, it is a construct and signification that is attributed to certain bodies in certain context at certain moments in time.

6. I use ethnicity and race here interchangeably, not to argue that they are the same, but to highlight how US-American discourses use ethnicity to delegitimize debates about white privilege and racism. These discourses do indeed use ethnicity in exchange for race. In the wake of Black Pride, white American minorities claimed their ethnicity to mark their position to reject their involvement in white hegemony and, by labeling African Americanness as ethnicity as well, delegitimizing claims of racism against people of color and Black Americans (Matthew Jacobson 2006, 20–22).

7. The historian Prothero (2016) describes the public fight for gaining national cultural hegemony between US-American conservatives and liberals from Jefferson to Gay Marriage.

8. Conservatives on the far right early on derided Melania with sexist and exoticizing, culturalizing, and sometimes racializing comments during the presidential campaign. Even the Trump-endorsing *New York Post* reprinted nude photographs of Melania from 1996 and 2001 (The Mirror 2016), that were then repeated or at least commented on by every major newspaper, mostly in derogatory language (Trebay 2016). In March 2016 a “conservative anti-Trump SuperPAC Make America Awesome,” ran “a series of extraordinarily low-budget, meme-like ads telling Mormons on Facebook and Instagram to vote for Ted Cruz” (Christina Cauterucci 2016). One of the ads used an image from a British *GQ* profile of Melania, then Donald Trump’s girlfriend, from 2000, where she lies naked on a fur rug, warning that she could be the next first lady. The liberal press is not much more prone to sympathize with the Slovenian-American First Lady and instead activates the most derogatory stereotypes about Eastern European women.

## References

[CIT0001] ABC News. 2016. “Meet the Artist behind the Yoruba Body Art in Beyonce’s ‘Lemonade’” *ABC News*. Accessed 427, 2016. http://abcnews.go.com/Entertainment/meet-nigerian-artist-yoruba-body-art-beyoncs-lemonade/story?id=38678748

[CIT0002] Anderson, Bridget. 2000. *Doing the Dirty Work? the Global Politics of Domestic Labor*, 152–154. London: Zed.

[CIT0003] Andersson, Eric. 2016. “Inside Melania Trump’s Life as a Mom: ‘I’m Very Involved.” *US Magazine*. Accessed 129, 2016. http://www.usmagazine.com/celebrity-news/news/inside-melania-trumps-life-as-a-mom-im-very-involved-w453982

[CIT0004] Aravosis, John. 2017. “Trump’s Treatment of Melania on Inauguration Day Was Abhorrent.” *America Blog*. Accessed 123, 2017. http://americablog.com/2017/01/way-trump-treated-melania-inauguration-day.html

[CIT0005] Arzumanova, Inna. 2016. “The Culture Industry and Beyoncé’s Proprietary Blackness.” *Celebrity Studies* 7 (3): 421–424. doi:10.1080/19392397.2016.1203613.

[CIT0006] Bruzzi, Stella. 2012. *Undressing Cinema: Clothing and Identity in the Movies*. New York/London: Routledge.

[CIT0007] Burleigh, Nina. 2017. “Melania, Ivanka, Ivana, Marla and the Role of Women in Trump’s World.” *Newsweek*. Accessed 21, 2017. http://www.newsweek.com/2017/02/10/melania-ivanka-ivana-marla-women-trump-world-550804.html

[CIT0008] Cauterucci, Christina. 2016. “There’s No Need to Slut-Shame Melania in Political Ads. Trump Is Horrible Enough!” *Slate Magazine*. Accessed 322, 2016. http://www.slate.com/blogs/xx_factor/2016/03/22/anti_trump_ads_try_to_win_over_mormons_by_slut_shaming_melania.html

[CIT0009] Ceric, Irina, Ana Grujic, Jasmina Tumbas, and Bojana Videkanic. 2016. “An Open Letter to the Editors of the Monkey Cage Blog of the Washington Post Online Edition.” *Balkanist*. Accessed 724, 2016. http://balkanist.net/an-open-letter-to-the-editors-of-the-monkey-cage-blog-of-the-washington-post-online-edition/

[CIT0010] Collins, Lauren. 2016. “The Model American: Melania Trump Is the Exception to Her Husband’s Nativist Politics.” *The New Yorker*. Accessed 59, 2016. http://www.newyorker.com/magazine/2016/05/09/who-is-melania-trump

[CIT0011] Crocker, Lizzie. 2016. “Why Aren’t Feminists up in Arms about the Slut-Shaming of Nude Melania Trump?” *The Daily Beast*. Accessed 82, 2016. http://www.thedailybeast.com/articles/2016/08/02/why-aren-t-feminists-up-in-arms-about-the-slut-shaming-of-nude-melania-trump.html

[CIT0012] Deltcheva, Roumiana. 2004. “Eastern Women in Western Chronotypes: Representation of East European Women in Western Film after 1989.” In *Vampirettes, Wretches and Amazons: Western Representations of East European Women*, edited by Valentina Glajar and Domnica Radulescu, 160–186. New York: Columbia University Press.

[CIT0013] Dwyer, Kate. 2017. “Melania Trump Was Recently Spotted in European Designers.” *Motto Time*. Accessed 28, 2017. http://motto.time.com/4664481/melania-trump-spotted-european-designers/

[CIT0014] ETN. 2013. “Donald Trump and Melania Knauss, 24-Year Age Difference.” *Earn the Necklace*. Accessed 612, 2016. https://www.earnthenecklace.com/donald-trump-and-melania-knauss-24-year-age-difference/

[CIT0015] Filipovic, Jill. 2017. “#Freemelania Is Sexist and Patronizing: She’s an Adult Woman, Not an Injured Little Bird.” *Cosmopolitan*. Accessed 130, 2017. http://www.cosmopolitan.com/politics/a8653896/melania-trump-freemelania-sexism/

[CIT0016] Friedman, Vanessa. 2017. “Melania Trump Reappears, and Wears European First.” *The New York Times*. Accessed 27, 2017. https://www.nytimes.com/2017/02/07/fashion/melania-trump-reappears-and-wears-european-first.html

[CIT0017] Frisby, C. M., and J. S. Aubrey. 2012. “Race and Genre in the Use of Sexual Objectification in Female Artists’ Music Videos.” *Howard Journal of Communications* 23 (1): 66–87. doi:10.1080/10646175.2012.641880.

[CIT0018] Glajar, Valentina, and Domnica Radulescu, eds. 2004. *Vampirettes, Wretches and Amazons: Western Representations of East European Women*. NY: Columbia University Press.

[CIT0019] Graham, David A. 2016. “Melania Trump’s Secret Speechwriter: Michelle Obama?” *The Atlantic*. Accessed 719, 2016. https://www.theatlantic.com/politics/archive/2016/07/melania-trump-plagiarism/491918/

[CIT0020] Hall, Stuart. 1997 “Race, The Floating Signifier.” *Transcript: Sut Jhalley, Media Education Foundation*. Accessed 721, 2015, http://www.mediaed.org

[CIT0021] Jacobson, Matthew. 2006. *Roots Too: White Ethnic Revival in Post-Civil Rights America*. Cambridge: Harvard University Press.

[CIT0022] Krieg, Gregory, Eric Bradner, and Eugene Scott. 2016. “No One to Be Fired after Melania Trump Speech Plagiarism Episode.“ *CNN*. Accessed 719, 2016. http://www.cnn.com/2016/07/19/politics/melania-trump-michelle-obama-speech/;

[CIT0023] Kulpa, Robert. 2014. “Western Leveraged Pedagogy of Central and Eastern Europe: Discourses of Homophobia, Tolerance, and Nationhood.” *Gender, Place & Culture,* 21 (4): 431–448. doi: 10.1080/0966369X.2013.793656

[CIT0024] Kulpa, Robert, and Joanna Mizielińska. 2011. *De-Centring Western Sexualities: Central and Eastern European Perspectives*. Farnham: Ashgate.

[CIT0025] Lazar, Michelle M. 2007. “Feminist Critical Discourse Analysis: Articulating a Feminist Discourse Praxis1.” *Critical Discourse Studies* 4 (2): 141–164. doi:10.1080/17405900701464816.

[CIT0026] The Mirror. 2016. “Donald Trump’s Wife Melania Naked Shoot for GQ Magazine as Girl-On-Girl Photos are Exposed.” *The Mirror*. Accessed 81, 2016. http://www.mirror.co.uk/3am/celebrity-news/donald-trumps-wife-melania-naked-8542914

[CIT0027] Nalepa, Monika. 2016. “Melania Trump and the Culture of Cheating in Eastern European Schools.” *The Washington Post*. Accessed 720, 2016. https://www.washingtonpost.com/news/monkey-cage/wp/2016/07/20/melania-trump-and-the-culture-of-cheating-in-eastern-european-schools/?utm_term=.1600d1173660

[CIT0028] Obeidallah, Dean. (2017). “SNL”: Merry Christmas, Here’s Your Next Culture War (Opinion). Accessed 425, 2018. https://www.cnn.com/2017/10/15/opinions/snl-trump-christmas-opinion-obeidallah/index.html

[CIT0029] Opatich, Jessica. 2016. “Beyoncé’s Masterpiece on the Black Experience.” *The Stony Brook Press*. Accessed 424, 2016. http://sbpress.com/2016/04/beyonces-masterpiece-on-the-black-experience/

[CIT0030] Parvulescu, Anca. 2014. *The Traffic in Women’s Work: East European Migration and the Making of Europe*. Chicago and London: University of Chicago Press.

[CIT0031] Perrott, Lisa, Holly Rogers, and Carol Vernallis. 2016. “Beyoncé’s Lemonade: She Dreams in Both Worlds.” *Film International*. Accessed 62, 2016. http://filmint.nu/?p=18413

[CIT0032] Prothero, Stepehn. 2016. *Why Liberals Win the Culture Wars (Even When They Lose Elections): The Battles that Define America from Jefferson’s Heresies to Gay Marriage*. New York: HaperCollins.

[CIT0033] Prtoric, Jelena. 2017. “Melania Trump: Is the First Lady a Victim of Anti-Eastern European Prejudice?” *The Calvert Journal: A Guide to the New East*. Accessed December 1, 2018. http://calvertjournal.com/comment/show/7575/melania-trump-new-east-first-lady

[CIT0034] Pyzik, Agata. 2016. “Why They Hate Melania Trump.” *Jakobin*. Accessed 815, 2016. https://www.jacobinmag.com/2016/08/melania-trump-donald-eastern-european-stereotypes/

[CIT0035] Robinson, Joanna. (2017). Is There a Reckoning Coming for Saturday Night Live? *Vanity Fair*. Accessed 425, 2018. https://www.vanityfair.com/hollywood/2017/10/saturday-night-live-trump-ratings-taran-killam-hypocrisy

[CIT0036] Sadowski-Smith, Claudia. 2018. *New Immigrant Whiteness: Race, Neoliberalism, and Post-Soviet Migration to the United States (Nation of Nations)*. New York: NYU Press.

[CIT0037] Tlostanova, Madina. 2015. “Can the post-Soviet Think? on Coloniality of Knowledge, External Imperial and Double Colonial Difference.” *Intersections* 1 (2). doi:10.17356/ieejsp.v1i2.38.

[CIT0038] Trebay, Guy. 2016. “Melania Trump, the Silent Partner.” *The New York Times*. Accessed 930, 2015. D1.

[CIT0039] Tuszynska, Agnieszka. 2004. “Eastern Girls, Western Boys: The Image of Eastern European Women in the Birthday Girl.” In *Vampirettes, Wretches and Amazons: Western Representations of East European Women*, edited by Valentina Glajar and Domnica Radulescu, 203–214. New York: Columbia University Press.

[CIT0040] Vernallis, Carol. 2016. “Beyoncé ’ S Lemonade, Avant-Garde Aesthetics, and Music Video: ‘The past and the Future Merge to Meet Us Here’.” *Film Criticism*, 2.

[CIT0041] Waguespack, Andrea. 2016. “Beyonce Releases ‘Sorry,’ the First Video from the Visual Album “Lemonade”. *Houston Chronicle*. Accessed 622, 2016. http://www.chron.com/entertainment/article/Beyonce-releases-Sorry-the-first-video-from-8319122.php

[CIT0042] Weaver, Alaura. 2017. “Melania, Are You Okay?” *Athena Talks*. Accessed 124, 2017. https://medium.com/athena-talks/melania-are-you-okay-5e7eadef2c5#.8jxkfxt8z

[CIT0043] Williams, Kimberly A. 2012. *Imagining Russia: Making Feminist Sense of American Nationalism in U.S.-Russian Relations*. Albany: State University of New York Press.

[CIT0044] Wolff, Larry. 1994. *Inventing Eastern Europe: The Map of Civilization on the Mind of the Enlightenment*. Stanford, CA: Stanford University Press.

[CIT0045] Worley, Will. 2017. “Melania Trump Just ‘An Object’ to Her Husband, Body Language Expert Says.” *The Independent*. Accessed 124, 2017. http://www.independent.co.uk/news/world/americas/melania-trump-first-lady-just-an-object-donald-trump-body-language-expert-us-president-white-house-a7542806.html

[CIT0046] Yoder, Katie. 2017. “Feminist Media Slam NYT: Melania a ‘Slut-Shaming Enabler,’ Not ‘Hooker’” *MRC News Busters*. Accessed 217, 2017. http://www.newsbusters.org/blogs/culture/katie-yoder/2017/02/17/feminist-media-slam-nyt-melania-slut-shaming-enabler-not-hooker

